# Analysis of genetic and nongenetic factors influencing triglycerides-lowering drug effects based on paired observations

**DOI:** 10.1186/s12919-018-0153-6

**Published:** 2018-09-17

**Authors:** Zheng Xu, Qing Duan, Juan Cui, Yumou Qiu, Qidong Jia, Cong Wu, Jennifer Clarke

**Affiliations:** 1 Department of Statistics, University of Nebraska, 340 Hardin Hall North Wing, Lincoln, NE 68588 USA; 2 Department of Food Science and Technology, University of Nebraska, 1901 N. 21st Street, Lincoln, NE 68588 USA; 3 Department of Genetics, University of North Carolina, 120 Mason Farm Road, Chapel Hill, NC 27599 USA; 4 Department of Computer Science and Engineering, University of Nebraska, 256 Avery Hall, Lincoln, NE 68508 USA; 5 Center for Biotechnology, University of Nebraska, 1901 Vine Street, Lincoln, NE 68588 USA; 6 Jiangxi Provincial Key Laboratory for Animal Health and Institute of Animal Population Health, College of Animal Science and Technology, Jiangxi Agricultural University, Nanchang, 330045 Jiangxi China

## Abstract

Obesity is a risk factor for heart disease, stroke, diabetes, high blood pressure, and other chronic diseases. Some drugs, including fenofibrate, are used to treat obesity or excessive weight by lowering the level of specific triglycerides. However, different groups have different drug sensitivities and, consequently, there are differences in drug effects. In this study, we assessed both genetic and nongenetic factors that influence drug responses and stratified patients into groups based on differential drug effect and sensitivity. Our methodology of investigating genetic factors and nongenetic factors is applicable to studying differential effects of other drugs, such as statins, and provides an approach to the development of personalized medicine.

## Background

Obesity and excessive weight (body mass index > 25) are highly prevalent among US adults and youth [1]. Obesity puts a person at a higher risk for heart disease, stroke, diabetes, high blood pressure, and other medical ailments. Consequently, effective treatment strategies for obesity and excessive weight designed to improve an individual’s health and quality of life are highly desired.

Genetic and nongenetic factors jointly influence the likelihood of obesity and being overweight [1, 2]. Obesity is associated with changes in blood lipid levels, which can increase the risk of cardiovascular diseases. Fenofibrate is recommended because of its triglyceride-lowering effect. However, many genetic and nongenetic factors may influence the effects of such medications. Instead of the same types and amounts of drugs for all patients, personalized medicine considers the differences in drug effects between individuals and recommends the optimal treatment strategy for each patient individually.

Our goal was to provide a better understanding of drug mechanisms and to contribute to the development of precision medicine by studying the genetic and nongenetic factors that influence the effects of fenofibrate in treatment of obesity. We identified groups of individuals with differential drug effects. In addition, our methodology has the potential to be applied in the study of differences in drug effects and personalized medicine based on other medicines such as statins.

## Methods

GAW20 provided the data. We are interested in understanding factors that may influence the drug effects of fenofibrate on triglyceride levels.

Our proposed methodology can work for the *paired observation* situation in which the research problem is how multiple factors influence the differences in drug effects. We measured the phenotypes of the *same individuals before and after* treatment with fenofibrate, and genotypes and nongenetic data on these individuals are available.

For a data set with paired observations, denote the response or phenotype of interest (drug effect in this context) before and after treatment with a drug as *Y*_*pre*_ and *Y*_*post*_. The change ∆*Y* = *Y*_*post*_ − *Y*_*pre*_ is the “drug effect.” In this study, our interest is the raw difference in the level of triglycerides at visit 4 (after the treatment) minus at visit 2 (before the treatment).

We first inspected the distribution of the drug effect (ie, response) ∆*Y* to characterize its average and variability. Then, among *s* nongenetic factors *C*_1_, *C*_2_, …, *C*_*s*_ and the top 10 principal components (PCs) of the genotypes *PC*_1_, …, *PC*_10_ (ie ancestry variables), we checked the association of these variables with drug effect in a multiple regression framework. When there are different genetic marker frequencies and different drug effects, there are drug–ancestry interactions that influence the drug effects.

It was noted that factors influencing phenotype *Y* may not be the factors influencing drug response ∆*Y*. For example, assume that before and after the treatment, factor *Z* influences *Y*. We can model *Y*_*pre*_ = *β*_0, *pre*_ + *β*_*Z*, *pre*_*Z* + …and *Y*_*post*_ = *β*_0, *post*_ + *β*_*Z*, *post*_*Z* + …. Then we have ∆*Y* = *Y*_*post*_ − *Y*_*pre*_ = (*β*_0, *post*_ − *β*_0, *pre*_) + (*β*_*Z*, *post*_ − *β*_*Z*, *pre*_)*Z* + …. For *Z* to be a factor influencing ∆*Y*, the factor Z has to have different magnitude in its effect on *Y*_*post*_ and *Y*_*pre*_, which is the drug-by-factor-*Z* interaction effect in the expression of *Y*. This interaction effect captures, for example, the situation in which *Z* has no effect on *Y*_*pre*_ but does affect *Y*_*post*_.

We assessed genetic variants genome-wide to find single-nucleotide polymorphisms (SNPs) associated with ∆*Y*. We divided the sets of SNPs into common SNPs and rare SNPs. For common SNPs (minor allele frequency [MAF] ≥5%), we conducted genome-wide association studies based on familial data, controlling for covariates. For rare SNPs (1% ≤ MAF < 5%), we conducted both gene-based and region-based rare variant tests based on familial data, using the fast family-based sequencing kernel association testing method (FFBSKAT), which is a specific method to extend the sequence kernel association test (SKAT) for unrelated individuals to familial data [3]. The FFBSKAT method was implemented using the Family REGional Association Tests (*FREGAT*) R package [4].

## Results

### Drug effects are triglyceride-lowering on average but with big variations

There are 1105 participants with phenotypes and covariates available, 4151 participants with pedigree information available, and 822 participants with genotypes available for a dense set of 718,542 SNPs from the Genetics of Lipid Lowering Drugs and Diet Network (GOLDN) study in the GAW20 data [5]. Our quality control (QC) step filtered out SNPs and individuals with a success rate of less than 97%, leaving 822 persons and 700,763 SNPs after QC. Note that the maximum genotype missing rate for an individual is 2.93% so that all 822 participants passed QC. The intersection of the 822 participants with genotypes, the 1105 participants with phenotype and covariates, and the 4151 participants with pedigree information includes 821 common individuals. Note that these 821 individuals have missing values in genotypes, phenotypes, and covariates, and we *did not* restrict our analysis only to the individuals with complete data. The 821 individuals are from 173 families. Thus, they are related individuals (familial data). We conducted analysis using a linear mixed model considering the relatedness within families. Nongenetic covariates include gender, age, field center (Minnesota and Utah), smoking status (never, past, and current smoker), metabolic syndrome defined by the adult treatment panel (ATP), and metabolic syndrome defined by the International Diabetes Federation (IDF) in the GAW20 [5].

**Fig. 1:**
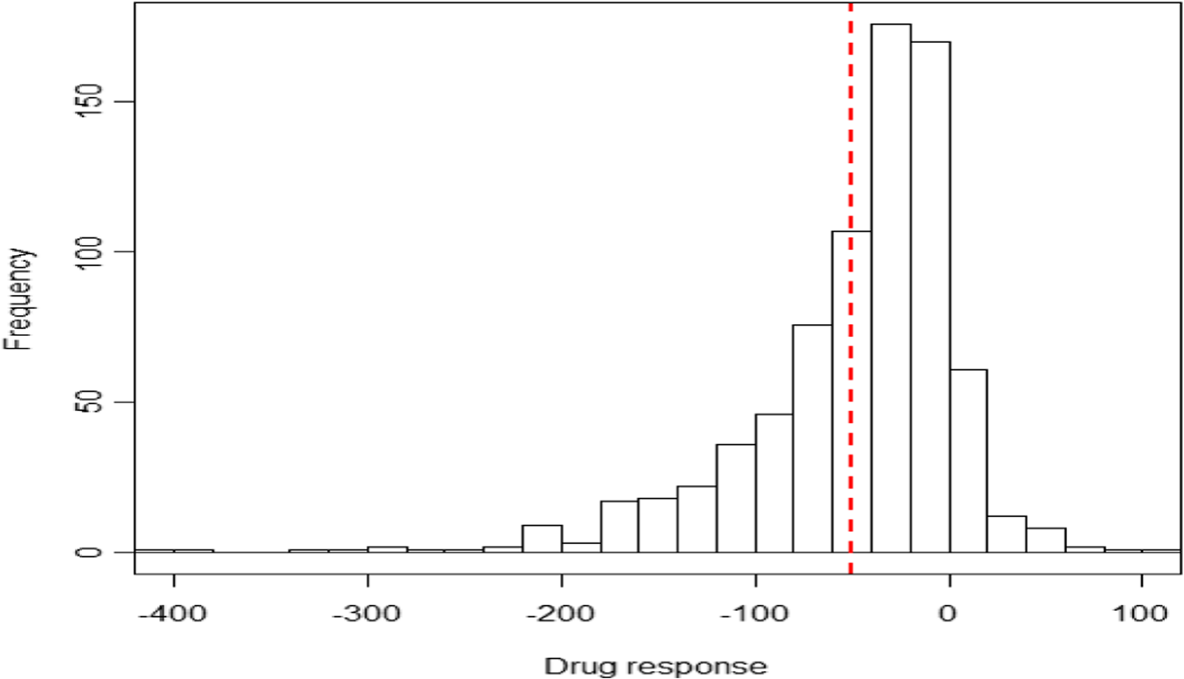
Histogram of drug responses

Figure 1 shows a histogram of drug response ∆*Y* (ie, changes in the level of triglycerides). We found there was a 50.37 mg/dL decrease on average, indicating the overall drug effect is triglyceride-lowering. However, there was a big variation in drug response ∆*Y*, implying differential drug effects.

### Nongenetic factors and ancestry

We next studied the effects of nongenetic factors and ancestry, that is, population structure. Ancestry was represented by the top 10 principal components (PCs) of genotypes at independent SNPs. We first used PLINK to prune SNPs based on linkage disequilibrium to generate a set of independent SNPs using the default setting, that is, squared correlation of < 0.1 [6]. Then we used the eigenstate software to calculate PCs of genotypes [7].

We conducted *multiple regressions* with more than one covariate considering that pairwise analyses (eg, the analysis of a drug response and only one covariate) suffer from confounding effects, cannot control for other covariates, and are less reliable. The regression of drug effects on nongenetic factors (age, center, gender, smoking, IDF, and ATP) and ancestry (PC_1_ to PC_10_) was conducted. Linear mixed-model–based testing was used because of the relatedness of individuals in familial data that was implemented using the *FREGAT* package of R software. A theoretical kinship matrix was calculated from pedigree information using the R (version 3.3.1) package *kinship2* [8]. We found statistical significance in center (*p* value = 0.013) and ATP (*p* value = 1.88 × 10^− 5^), but no significance in age (*p* value = 0.053), gender (*p* value = 0.126), smoking (*p* value = 0.067), IDF (*p* value = 0.137), or PC_1_ to PC_10_. These findings, especially the insignificance of PC_1_ to PC_10_, were consistent with the results of other GAW20 groups, even though different analysis frameworks were used.

### Genome-wide association study of common SNPs

**Fig. 2:**
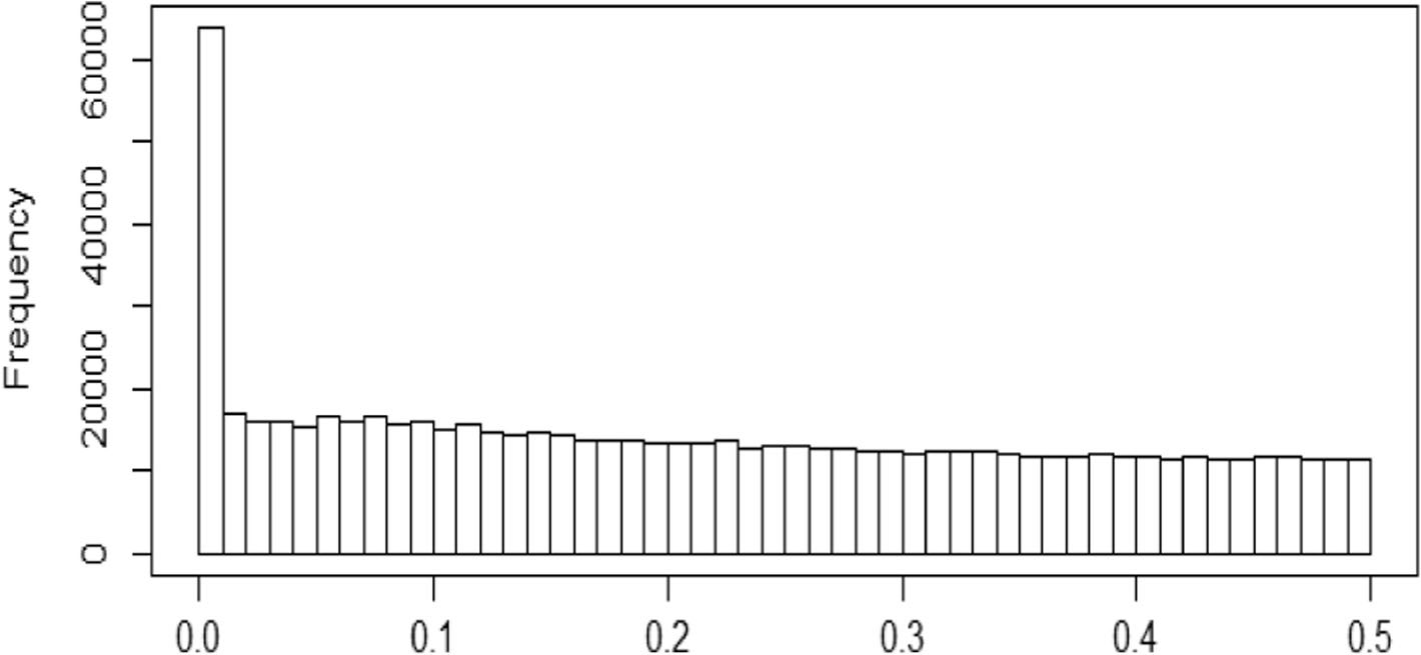
Histogram of MAFs

**Fig. 3:**
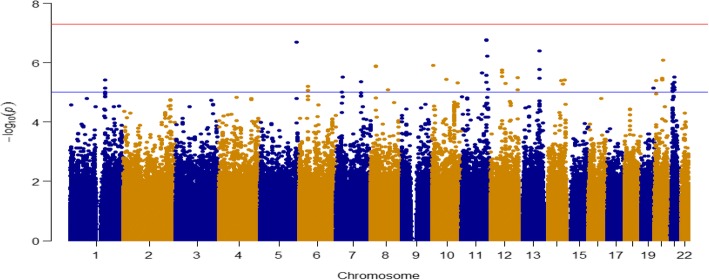
Manhattan plot for common SNPs

We divided SNPs with a MAF ≥ 0.01 into common and rare SNPs. Figure 2 is a histogram of MAFs. Because we have only 821 related individuals from 173 families, making the effective sample size smaller than 821, we considered SNPs with a MAF ≥ 0.05 as common, and SNPs with 0.01 ≤ MAF < 0.05 as rare [9]. There are 574,602 common SNPs in our analysis. We conducted a genome-wide association study (GWAS) for common SNPs for familial data, implemented using the R package GWAF [10]. We converted *p* values into false discovery rate (FDR) *q* values using Benjamini and Hochberg’s method, implemented in the R package fdrtool [11, 12]. We controlled for an FDR ≤ 0.05 in our report. Figure 3 shows the Manhattan plot and Table 1 lists the top 10 SNPs.

**Table 1:** Top 10 SNPs in GWAS for common SNPs

Chr	Base pair	SNP name	*p* value	*q* value	FDR < 5%
11	116,154,127	rs964184	1.73E-07	3.74E-02	Yes
11	116,208,850	rs5128	1.76E-07	3.74E-02	Yes
5	170,899,076	rs919758	2.02E-07	3.74E-02	Yes
13	96,316,286	rs9516776	3.91E-07	4.84E-02	Yes
11	119,259,136	rs503175	6.05E-07	5.44E-02	No
20	37,617,241	rs4812401	8.06E-07	5.77E-02	No
10	4,516,700	rs10795173	1.22E-06	6.14E-02	No
8	25,544,459	rs10081452	1.29E-06	6.19E-02	No
8	25,549,340	rs1425739	1.32E-06	6.21E-02	No
13	96,315,120	rs7332653	1.68E-06	6.37E-02	No

### Gene-based and region-based rare-variant association testing results

**Fig. 4:**
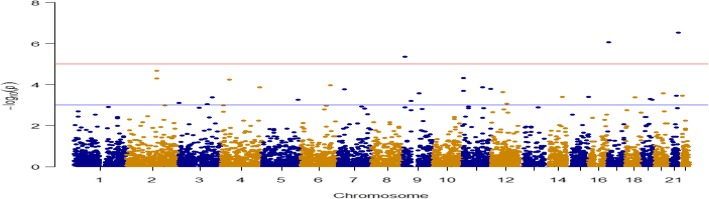
Manhattan plot for gene-based rare-variant testing

We used the R package *FREGAT* to conduct gene-based and region-based rare-variant association testing based on familial data [3, 4]. There are 63,689 rare SNPs (ie, 0.01 ≤ MAF < 0.05) in our analysis. We downloaded gene annotations (UCSC build hg19) including 57,816 genes. SNPs that lie within 1 kb of the flanking region upstream and downstream of each gene were considered as promoters are usually within 1 kb of the associated gene transcription start site; 6138 genes included at least one rare SNP for testing. Figure 4 shows a Manhattan plot using the midpoints of genes as base pair locations and Table 2 lists the top 10 genes. There are 6 genes with an FDR < 0.05, namely, *DNMT3L, SPATA22, RP11-403H13.1, AC010740.1, OR52N4,* and *LRP1B*.

**Table 2:** Top 10 genes in gene-based rare-variant test

Gene	Chr	Base pair of midpoint	*p* value	*q* values	FDR < 5%
*DNMT3L*	21	45,674,160	2.97E-07	1.81E-03	Yes
*SPATA22*	17	3,380,229	8.71E-07	2.66E-03	Yes
*RP11-403H13.1*	9	6,940,764	4.31E-06	8.76E-03	Yes
*AC010740.1*	2	141,656,333	2.14E-05	3.06E-02	Yes
*OR52N4*	11	5,776,441	4.74E-05	4.67E-02	Yes
*LRP1B*	2	141,939,131	4.94E-05	4.75E-02	Yes
*RP11-722 M1.1*	4	36,570,840	5.84E-05	5.09E-02	No
*AHI1*	6	135,711,792	1.07E-04	7.10E-02	No
*FAM76B*	11	95,512,839	1.33E-04	7.81E-02	No
*NEIL3*	4	178,257,543	1.35E-04	7.88E-02	No

**Fig. 5:**
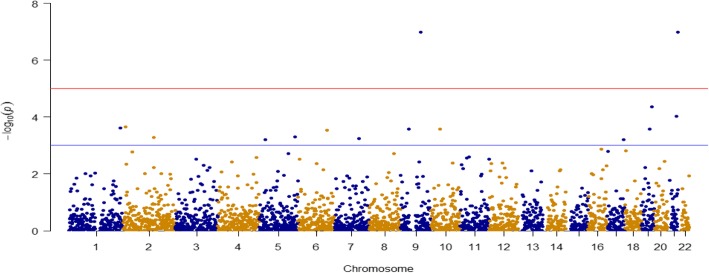
Manhattan plot for region-based rare-variant testing

We also conducted a region-based rare-variant test with every 1 Mb as 1 region. The whole genome was divided into 2686 regions. Figure 5 shows a Manhattan plot using the midpoint of regions as base pair locations and Table 3 lists the top 10 regions. There are 3 regions with an FDR ≤ 0.05. They are Chr9: 94 M–95 M, Chr21: 45 M–46 M, and Chr19: 49 M–50 M. It is encouraging that our previously discovered gene *DNMT3L* (Chr21: 45,666,222-45,682,099) also lies in our reported range Chr21: 45 M–46 M.

**Table 3:** Top 10 regions in region-based rare-variant test

Chr	Start base pair	End base pair	*p* value	*q* value	FDR < 5%
9	94M	95M	1.01E-07	1.36E-04	Yes
21	45M	46M	1.02E-07	1.36E-04	Yes
19	49M	50M	4.40E-05	3.66E-02	Yes
21	39M	40M	9.59E-05	5.53E-02	No
2	11M	12M	2.17E-04	7.30E-02	No
1	238M	239M	2.45E-04	7.52E-02	No
10	42M	43M	2.64E-04	7.64E-02	No
9	38M	39M	2.67E-04	7.66E-02	No
9	37M	38M	2.68E-04	7.67E-02	No

## Discussion

A lot of factors have the potential to influence drug responses, shown as a change in triglyceride levels, to fenofibrate treatment. This study is based on a linear mixed-model multiple regression (1) for nongenetic factors and ancestry variables, (2) for common SNPs, and (3) for rare SNPs. These analyses were performed using the R packages *FREGAT* and *GAWF*, with sample relatedness represented by theoretical kinship matrix, calculated using the R package *kinship2*.

Our analysis is based on 821 persons from 173 families. The effective sample size of this familial data is smaller than 821; consequently, there may not be enough power to identify associated variants. In addition, our GWAS analysis for common SNPs was based on a set of 574,602 SNPs without imputation. Imputation of genotype or summary statistics may uncover more associated SNPs, thereby increasing power [13, 14].

Despite a relatively small effective sample size, we still found that for nongenetic factors, some variables had *p* values < 5%, some had *p* values between 5 and 10%, and other variables had *p* values > 10%; and that for SNPs, there are 4 SNPs, 6 genes, and 3 regions of 1 Mb reported with an FDR controlled at 5%.

The roles of the top SNP, rs964181, and the top gene, *DNMT3L*, were also found in other published studies of obesity and triglyceride levels. The top SNP, rs964184, was found to be associated with hypertriglyceridemia [15], as well as with a lipid-lowering response to another medicine, statins [16]. The top gene, *DNMT3L*, is an enzymatically inactive regulatory factor, regulates DNA methylation activity, and is closely associated with epigenetic functions influencing obesity from epigenetic and regulation evidence [17]. *DNMT3L* encodes a DNA (cytosine-5)-methyltransferase 3–like enzyme, and an increased expression of DNA methyltransferase is found in obese adipose tissue [18]. A DNA methylation study revealed differential modification of many obesity genes before and after gastric bypass and weight loss, providing a model to investigate obesity and weight loss in humans [19].

The above association results only suggest and prioritize potential factors for future biological verification. Some reported significant variables may be just false positives. Following statistical analyses, functional analyses via biologically experimental verification and additional support from the published literature are needed. Integrative genome browsers with the database of GWAS catalog, gene annotations, and epigenetic and regulatory information can be used for this purpose [20, 21].

## Conclusions

We conducted an assessment of nongenetic and genetic factors that impact the drug response, shown as a change in triglyceride level, to fenofibrate treatment based on the GOLDN study data, and identified groups of participants with different drug sensitivities. We report significant associations of drug response with center and ATP variables with *p* values less than 5%, and 4 common SNPs (rs964184, rs5128, rs919758, and rs9516776), 6 genes (*DNMT3L*, *SPATA22*, *RP11-403H13.1*, *AC010740.1*, *OR52N4*, and *LRP1B*) and 3 regions of 1 Mb (Chr9: 94 M–95 M, Chr21: 45 M–46 M, and Chr19: 49 M–50 M) at an FDR controlled at 0.05. It is also encouraging that the reported gene *DNMT3L* (Chr21: 45,666,222-45,682,099, from a gene-based test) also lies in our reported range of Chr21: 45 M-46 Mb (from a range-based test). The roles of the top SNP, rs964184, and the top gene, *DNMT3L*, were also found in other studies on obesity and triglycerides. Both gene-based and region-based tests implied that *DNMT3L* plays a crucial role in influencing the mechanism and effects of triglyceride-lowering drugs treating obesity. Our methodology can be applied to studying other drugs, such as statins, and provides an approach to the development of personalized medicine.
